# Clinical Report

**Published:** 1898-06

**Authors:** Keating Bauduy


### CLINICAL REPORT.

BY KEATING BAUDUY, M.D.

CASE 1.—Mrs. S., aged thirty-two years, mother of three children, came to me in a pitiable mental condition, and had in her arms a nursing hydrocephalic child, five months old. Her mental drepression approached a type of veritable melancholia. My first idea was to advise that the child be weaned, and then place her upon the classical opium treatment for melancholia. This was her third child, and like all mothers, she clung to the life of her unfortunate with characteristic tenderness. Therefore, she bluntly insisted upon my candid opinion as to whether the weaning of the baby might prove fatal. Knowing, as I did, that the life of the child was simply a question of a period of short duration in either case, I so informed her; nevertheless, I insisted that the best hope for her recovery was to wean it. This she refused to do, and after Dr. Fisch had made a blood examination and prononnced her highly anæmic, I reluctantly undertook the case. Aside from her mental depression, physical lassitude and marked pallor, the "casque neurasthenique" symptom was a dominant feature in her case. Any effort to perform her usual household duties produced sensations of cerebral fullness and persistent pain in the vertex. She even confessed that the idea of suicide had of late frequently haunted her. Under the administration of "Pepto-Mangan," with no other treatment, after the short period of fifty-two days she was discharged, fully restored to her normal condition. Microscopic report showed a relative gain in number of red blood corpuscles of 34 per cent.; hemoglobin, 44.5 per cent.

CASE 2.—Mrs. Sim, age twenty-three years, mother of two children, youngest six months and nursing. About the fourth month of her last pregnancy she was troubled with dyspnœa. Gave history of instrumental delivery, followed by puerperal eclampsia. Great loss of blood during birth of child. Two months later abscesses developed in each breast and patient was confined to bed during a period of ten weeks. Case presented typical manifestations of neurasthenia, also characteristic apprehensions, with preternatural emotional mobility. Constant cephalalgia in vertical region, persistent parasthesiæ in extremities, mouth and tongue, were also present. She was intensely pale with every appearance of profound anæmia. Aside from a mild laxative which was given to obviate constipation—an obstinate feature in her case—nothing was administered save "Pepto-Mangan." After a period of treatment of forty-nine days I discharged her, as she evinced none of the symptoms which formerly existed. A notable feature was the corresponding improvement of the child, notwithstanding the fact that I had previously insisted upon its being weaned, which she had nevertheless, contrary to my instruction, continued to nurse. Microscopic report showed a relative gain: red blood corpuscles, 19 per cent.; hemoglobin, 27 per cent.

CASE 3.—D. G., aged twenty-five years, unmarried. Suffered from nervous headache for past year. Vaso-motor disturbances evidenced by alternate flushings and pallors, heat and cold. Atonic dyspepsia. Irregularity of bowels. Disturbed sleep. Depressed physical condition, correspondingly weak pulse. After taking "Pepto-Mangan" fifty-seven days reported feeling generally improved. Digestion was better, pulse stronger and headaches greatly diminished in intensity. Vaso-motor disturbances disappeared. Microscopic examinations showed a relative gain: red blood corpuscles, 11 per cent.; hemoglobin, 15 per cent.

CASE 4.—Miss S., aged twenty-eight years, presenting many of the well-defined symptoms of neurasthenia, was in a condition of profound mental and physical weakness. The history showed that since our great cyclone of May 27, 1896, she had never been her normal self, and was unable to perform any sustained mental or physical strain. Dating from that episode she had always worried and was constantly the victim of peculiar forebodings. Insomnia and general malaise were cardinal symptoms. My diagnosis was what has been termed "cyclone neurosis," of which I have seen numerous cases. Menorrhagia existed to an alarming extent, for which I accordingly recommended rest and the recumbent posture during her periods. Because of the pronounced insomnia, I prescribe a nightly dose of hyoscyamine and sulfonal during the first week of treatment as a hypnotic, which constituted the only medication other than "Pepto-Mangan." After having taken the latter for forty-one days I discharged her from treatment, as she had passed her last menstrual period after a normal flow of three days, her pallor having given way to rosy cheeks and her physical and mental condition being entirely satisfactory. Microscopic report showed a relative gain: red blood corpuscles, 38 per cent.; hemoglobin, 47 per cent.

CASE 5.—Mr. C., aged twenty-one years, unmarried. Highly anæmic, very pale. Anorexia and insomnia persistent. Physical condition greatly depressed. Cardinal feature was a sexual hypochondriacal tendency. Gave history of excesses both alcoholic and sexual. Aside from advice as to the necessity of leading a moral life and abstaining from all stimulants, gave no medicine but "Pepto-Mangan," with the addition of arsenic and strychnia. After fifty-seven days of treatment patient was much benefited. Microscopic report showed a relative gain: red blood corpuscles, 9 per cent.; hemoglobin, 27 per cent.

CASE 6.—Mrs. D., aged thirty-six years, married; five children. Since birth of last child, eighteen months ago, has been in state of profound nervous prostration. Previously resisted ordinary tonic and constructive treatment. Menorrhagia was the dominant feature of the case. After taking "Pepto-Mangan" for fifty-one days patient evinced more improvement than during any stated time throughout the past eighteen months. Last menstruation approached the normal flow. Microscopic report showed a relative gain: red blood corpuscles, 13 per cent.; hemoglobin, 8 per cent.

CASE 7.—Mrs. J., aged forty-eight years, widow; mother of a large family. Cardinal feature of case was recurrent cephalalgia at intervals of several days. This case reported an improvement as to the intensity and duration of headaches after the period of fourteen days of treatment. Only two blood examinations were made. A further opportunity to observe this patient did not present itself, in consequence of her failure to continue the treatment. Microscopic examination showed a relative gain: red blood corpuscles, 14 per cent.; hemoglobin, 13 per cent.

CASE 8.—H. F., aged eighteen years, school teacher, unmarried. Symptomatology of neurasthenia. Malaria was a complicating feature. Amenorrhœa for past six months was the principal symptom for which she consulted me. Aside from a course of quinine to eradicate the malarial feature, I exclusively gave "Pepto-Mangan." After forty-seven days' treatment she was apparently much improved, her menses having appeared in the interim. Microscopic examination showed a relative gain: red blood corpuscles, 9 per cent.; hemoglobin, 22 per cent.

CASE 9.—Mrs. L., aged forty-two years, married; three children. Comes from neuropathic family, one uncle an epileptic. Has always been quite delicate and anæmic. Since sudden death of husband has mainfested great irritability of temper. Loses control of herself upon the slightest provocation. Cries easily, but not melancholic. Peculiarly apprehensive of sudden death; imagines upon retiring that she will never awake; paroxysmal attacks of anxiety, and fatigued upon the slightest exertion. Anorexia. Habitual constipation. Sleeps restlessly. Patient, although still very pale, after taking "Pepto-Mangan" for twenty-seven days began to manifest a general improvement. Microscopic report showed a relative gain: red blood corpuscles, 11 per cent.; hemoglobin, 12 per cent.

CASE 10.—Mrs. P., aged thirty-six years, married, no children. Family history predisposed to tuberculosis. Physically in good health. Since cyclone, May 27, 1896, when her house was totally destroyed, and she narrowly escaped death, she developed nervous headaches; later on she manifested a listless and apathetic condition. Sleeps excellently, but does not feel refreshed upon awakening. Complains of drowsiness. Marked irritability of temper. Appetite fair, but nervous dyspepsia. Boards with sister, as she can not muster courage to manage a household of her own. After taking "Pepto-Mangan" for twenty-five days she began to feel much brighter and better, but still occasionally lapses into her former indifferent mood. Color better, and nervous dyspepsia greatly relieved. Microscopic report showed a relative gain: red blood corpuscles, 12 per cent.; hemoglobin, 12 per cent.

CASE 11.—Mr. M., aged twenty-nine years. Family history tuberculous. His avocation was that of a "book-maker" during the past few years. The strain of gambling and the consequent excitement and worry have made him a nervous wreck. Jerky and fidgety at all times. Inability to concentrate his mind any time. Suffers from nightmares and phantasmagoria during sleep, which is consequently much disturbed. Is troubled with constipation and greatly impaired digestion. Anorexia marked. Much reduced in weight. Although always fatigued and depressed, he constantly walks to relieve his pent-up nervous irritability. Dreads to be alone for fear something may happen to him. After the administration of "Pepto-Mangan" for twenty-four days, patient reports a general improvement, especially as to his appetite and the relief of his indigestion. Microscopic report showed a relative gain: red blood corpuscles, 11 per cent.; hemoglobin, 12 per cent.

CASE 12.—A. McG., aged twenty years, servant, unmarried. History showed the ordinary "symptom-group" of neurasthenia. After the short period of seven days, having taken but one bottle of "Pepto-Mangan," her condition was greatly alleviated. Microscopic report showed a relative gain: red blood corpuscles, 5 per cent.; hemoglobin, 8 per cent.

